# Change in prevalence of gestational diabetes and obstetric complications when applying IADPSG screening criteria in a Belgian French speaking University Hospital. A retrospective cohort study

**DOI:** 10.1186/s12884-019-2406-4

**Published:** 2019-07-16

**Authors:** Elena Costa, Christine Kirckpartick, Colette Gerday, Aricia De Kempeneer, Sara Derisbourg, An Vercoutere, Sophie Haumont, Axelle Pintiaux, Caroline Daelemans

**Affiliations:** 1 Department of Obstetrics and Gynaecology, Hôpital Erasme, Route de Lennik 808, 1070 Anderlecht, Belgium; 2 Department of Endocrinology, Hôpital Erasme, Route de Lennik 808, 1070 Anderlecht, Belgium

**Keywords:** Gestational diabetes Mellitus, IADPSG criteria, Two step criteria, Obstetric outcomes

## Abstract

**Background:**

In April 2012 our institution chose to switch from a two- step criteria for Gestational Diabetes Mellitus (GDM) screening, to the International Association of Diabetes in Pregnancy Study Group (IADSPG) criteria. This shift led to an increased prevalence of GDM in our pregnant population. We designed a study in order to estimate the magnitude of the increase in GDM prevalence before and after the switch in screening strategy. As a secondary objective we wanted to evaluate if there was a significant difference between the two periods in the percentage of maternal and neonatal complications such as gestational hypertensive disorders (GHD), primary cesarean section (pCS), preterm birth, large for gestational age (LGA) newborns, macrosomia, shoulder dystocia, 5′ Apgar score less than to 7 at birth, neonatal intensive care unit (NICU) transfer and neonatal hypoglycemia.

**Methods:**

We selected retrospectively 3496 patients who delivered between January 2009 and December 2011 who were screened with the two-step criteria (group A), and compared them to 2555 patients who delivered between January 2013 and December 2014 and who were screened with IADPSG criteria (Group B). We checked patients’ electronic files to establish GDM status, baseline characteristics (age, body mass index, nationality, parity) and the presence of maternal and neonatal complications.

**Results:**

GDM prevalence increased significantly from group A (3.4%; 95%CI 2.8–4.06%) to group B (16.28%; 95%CI 14.8 -17.7%). In group B there were significantly more non-Belgian and primiparous patients. There was no statistically significant difference in maternal and neonatal complications between the two groups, even after adjustment for nationality and parity.

There was a non-significant reduction of the proportion of macrosomic and of LGA babies.

**Conclusions:**

In our population the introduction of IADPSG screening criteria has increased the prevalence of GDM without having a statistically significant impact on pregnancy outcomes.

## Background

The World Health Organization (WHO) in 1999 has defined Gestational Diabetes Mellitus (GDM) as a carbohydrate intolerance resulting in hyperglycemia of variable severity, with onset or first recognition during pregnancy [1]. Extensive research has demonstrated that GDM is associated with short- and long-term complications concerning both mother and child. Screening and treating GDM are an effective means to prevent short term complications [2] and a significant opportunity for intervention in order to avoid long term ones [3] . Short term complications are related to excessive fetal size leading to increased risk of difficult labor and delivery [4–6] and to the occurrence of maternal hypertensive disorders [6]. Long term complications include maternal increased risk of developing type 2 diabetes (T2DM) later in life and major potential metabolic pattern disorders in the offspring, which would lead to an increased risk of abnormal glucose tolerance, obesity and metabolic syndrome [3]

The International Association of Diabetes in Pregnancy Study Group (IADPSG) in 2010, following the Hyperglycemia and Adverse Pregnancy Outcome (HAPO) Study [5], has set a strategy of GDM screening based on a universal one-step 75 g Oral Glucose Tolerance Test (OGTT) between 24 and 28 weeks of gestation (WG). This allowed identifying, on an arbitrary basis, an excess risk of 75% of neonatal weight, a concentration of C-Peptide in the umbilical cord and adiposity in the newborn, each or all above the 90th percentile.

Applying IADPSG criteria invariably increases the prevalence of GDM in a given population, since it includes milder cases of GDM [7–11].

Our institution, Hôpital Erasme, is the Academic Hospital of the Université Libre de Bruxelles (ULB), a tertiary referral center that serves mainly the southern boroughs of the city of Brussels and the Belgian province of Hainaut.

In our department we switched from a GDM screening strategy with two-step criteria (50 g Glucose Challenge Test (GCT) followed, if positive, by 75 g OGTT [12]) to the one-step IADPSG criteria in 2012. The switch was made after the GGOLFB (Groupement des Gynécologues Obstétriciens de Langue Française de Belgique), the Belgian association of French speaking obstetricians and gynecologists, endorsed IADPSG criteria for GDM screening [13].

The primary objective of this study is to estimate the difference in prevalence of GDM in the pregnant population screened in our institution according to one-step and two-step strategies.

Our secondary objective is to compare the frequency of the subsequent maternal and neonatal outcomes that are usually associated with GDM, before and after we changed screening strategy, such as: Gestational Hypertensive Disorders (GHD), primary Cesarean Section (pCS), preterm birth, shoulder dystocia, macrosomia, Large for Gestational Age (LGA) newborns, Neonatal Intensive Care Unit (NICU) transfer and neonatal hypoglycemia.

## Methods

We designed a retrospective cohort study. We included all patients who delivered in our institution between January 2009 and December 2011 (Group A) and all patients who delivered between January 2013 and December 2014 (Group B). Patients in Group A were screened with two-step criteria: they underwent a 50 g Glucose Challenge Test (GCT) at 24 WG, then in the case the GCT was equal or above 140 mg/dl, the patients were tested with a 75 g Oral Glucose Tolerance Test (OGTT). They were considered positive if at least two out of three values were above the following thresholds: 95 mg/dl fasting, 180 mg /dl after 1 h, 155 mg/dl after two hours. Patients in Group B were screened using the one-step IADPSG criteria: they were given a Fasting Plasma Glucose test (FPG) at first visit, and diagnosed with GDM if it was equal to or greater than 92 mg/dl. The patients who had a negative FPG were screened between 24 and 28 WG with a 75 g OGTT that was considered positive if at least one value exceeded the thresholds (92 mg/dl fasting, 180 mg/dl at one hour, 153 mg/dl at two hours). We did not consider individuals who delivered in 2012, since this was a transition year in the screening strategy. We also excluded from analysis all patients who carried a multiple pregnancy. If the same patient delivered twice in the time frame defined by the study, we considered only the first delivery, in order to keep observations independent. We retrieved all data by extracting them from our electronic patient filing system. We excluded all patients for whom the results of the GDM screening test were not reported in the file. This included patients who were not screened in our institution (they were followed up in private practices and came to our institution only for delivery), patients who refused screening, patients who did not tolerate screening, and patients already followed up for pre-gestational diabetes.

In our institution all patients diagnosed with GDM were given dietary advice, taught to self-monitor glycaemia and were treated with medication if judged necessary by the endocrinologist. Glycemic targets were 95 mg/dl fasting and 120 mg/dl at 2 h postprandial. In the periods included in our study, diabetological care was coordinated by the same endocrinologist and glycemic targets did not change.

For each subject, we collected the following baseline data: age (divided into four categories: < 18 years, 18–29 years, 30–39 years and > 39 years), maternal pre-gestational Body Mass Index (BMI) (divided into four categories: underweight if < 18.5 Kg/m^2^, normal weight if 18.5–24.99 Kg/m^2^**,** overweight if 25–30 Kg/m^2^ and obese if > 30 Kg/m^2^), nationality at birth of the mother (divided into two categories: Belgian and non-Belgian), parity (divided into two categories: primiparous and multiparous) and previous CS (presence or absence of a cesarean section in the obstetric history of the multiparous patients). For each subject, we collected the following outcome variables: GDM screening results, presence of GHD (presence or absence of either gestational hypertension, preeclampsia or eclampsia), pCS (presence or absence of CS in the index pregnancy without previous history of CS), preterm birth (delivery before 37 WG), occurrence of shoulder dystocia (recording of one or more maneuvers aimed at resolving shoulder dystocia during delivery), macrosomia (presence or absence of newborn weight > 4000 g), LGA newborns (presence or absence of newborn weight > p90 for gestational age), 5′ Apgar score (divided in two categories > = 7 or < 7), transfer to NICU (hospitalization of the newborn in the NICU for any amount of time), and neonatal hypoglycemia (glycemia in the cord blood of the newborn < 50 mg/dl). We calculated percentiles of birth weight for newborns using AUDIPOG (Association des Utilisateurs de Dossiers Informatisés en Pédiatrie, Obstétrique et Gynécologie) curves [14]. Independently from the result of the screening test, the health practitioner had the possibility to flag the patient as affected by GDM.

This study has been approved by the independent ethics institutional review board (IRB) of Hôpital Erasme.

### Statistical analysis

We compared baseline characteristics of the two cohorts using the chi squared test. We computed prevalence of GDM for the two groups and we assessed 95% CI using the exact method [15]. We compared GDM prevalence and proportions of obstetrical complications using the chi squared test, then computed Odds Ratios (OR) and 95% CI using a univariate logistic regression. We corrected the crude OR by baseline characteristics which had a difference in the two groups of a *p*-value of 0.2 or less, by multivariate logistic regression, producing adjusted OR (aOR) and 95% CI.

We considered statistically significant a two-tailed *p*-value inferior to 0.05. We performed all statistical analysis using STATA 15 for Windows.

## Results

**Fig. 1:**
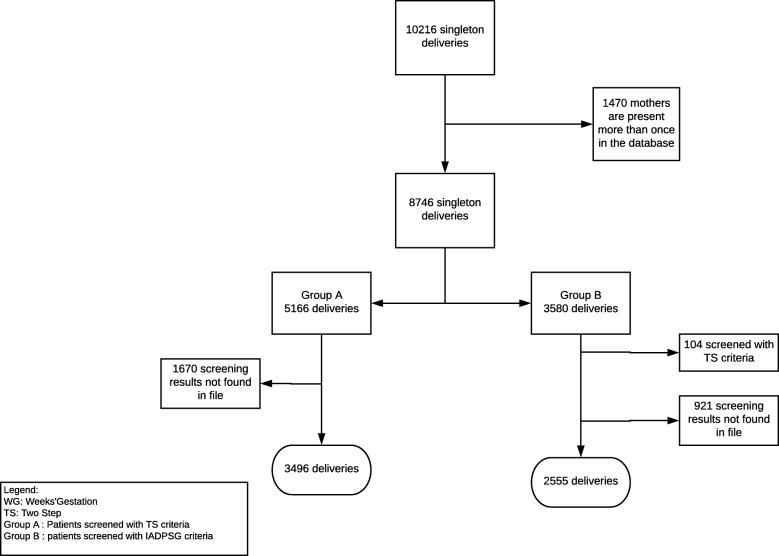
Flow chart of patients’ inclusion

We recruited 3496 subjects in Group A and 2555 subjects in Group B, the flow chart of patient selection is described in Fig. 1.

There was no statistically significant difference between the two groups in the distribution of maternal age and pre-gestational BMI. The proportion of Belgian patients was significantly higher in Group A (80.21%) compared to Group B (74.01%, *p*-value < 0.01). The proportion of multiparous patients was also higher in Group A (57.29%) compared to Group B (51.55%, *p*-value < 0.01). Table 1 illustrates baseline characteristics in the two groups.

**Table 1:** Baseline characteristics

	Group A	Group B	*p*-value
Age	*n* = 3496	*n* = 2555	0.705
< 18	14 (0.4%)	9 (0.35%)	
18–29	1668 (47.71%)	1184 (46.34%)	
30–39	1640 (46.91%)	1237 (48.41%)	
> 39	174 (4.98%)	125 (4.89%)	
Maternal BMI^a^	*n* = 2908	*n* = 2005	0.438
Underweight	126 (4.33%)	77 (3.84%)	
Normal Weight	1660 (57.08%)	1134 (56.87%)	
Overweight	737 (25.34%)	499 (24.89%)	
Obese	385 (13.24%)	295 (14.71%)	
Nationality	n = 3496	*n* = 2555	< 0.01
Belgian	2804 (80.21%)	1891 (74.01%)	
Non-Belgian	692 (19.79%)	664 (25.99%)	
Parity	n = 3496	n = 2555	< 0.01
Primiparous	1493 (42.71%)	1238 (48.45%	
Multiparous	2003 (57.29%)	1317 (51.55%)	

^a^Missing values maternal BMI 16.7%, randomly distributed in age, nationality, parity and GDM

**Fig. 2:**
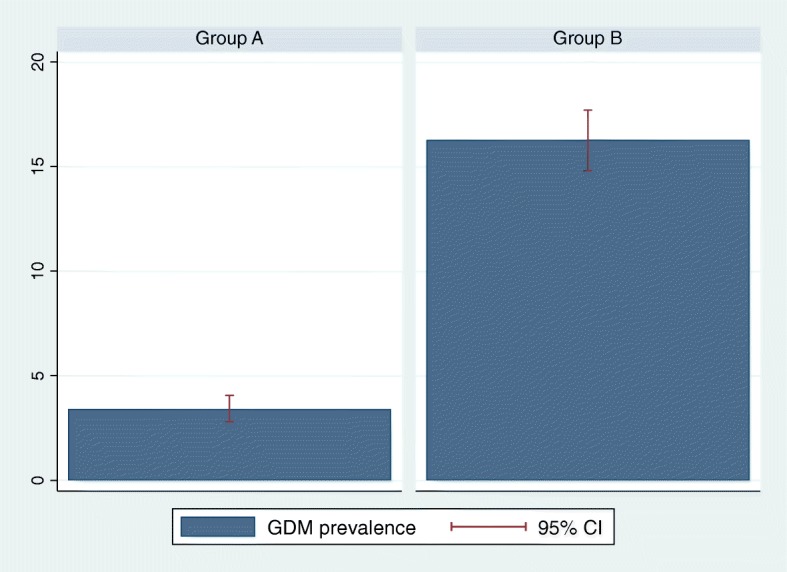
Prevalence of GDM patients in Group A and B

In Group A, 119 out of 3496 tests were positive for GDM (3.4%; 95%CI 2.8–4.06%), in Group B, 416 out of 2555 (16.3%; 95%CI 14.8 -17.7%) which translates into an OR of 5.51 (95%CI 4.46–6.81, *p*-value < 0.01). The difference in prevalence is illustrated in Fig. 2.

**Fig. 3:**
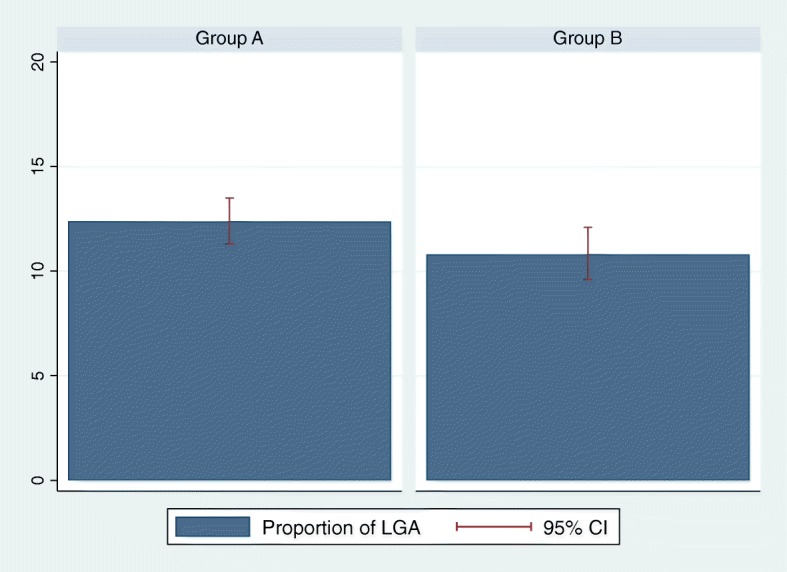
Prevalence of LGA newborns in Group A and B

**Fig. 4:**
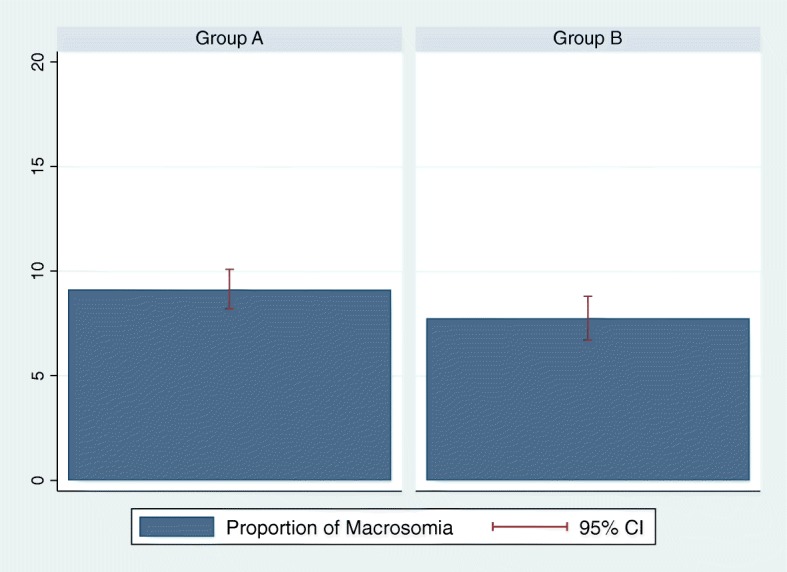
Prevalence of macrosomia in Group A and B

The proportion of LGA babies was lower in Group B (10.81%; 95%CI 9.6–12.1%) compared to Group A (12.39%; 95%CI 11.3–13.5%), as illustrated in Fig. 3, but the difference did not reach statistical significance (*p*-value 0.059). The proportion of macrosomia was also non-statistically significantly lower in Group B (7.75%; 95%CI 6.7–8.8%) compared to Group A (9.12%; 95%CI 8.2–10.1%; p-value 0.059), as can be seen in Fig. 4. With the exception of the prevalence of GDM, all the outcomes analyzed (LGA, macrosomia, 5’Apgar score, GHD, preterm birth, pCS, transfer to NICU and neonatal hypoglycemia) did not differ significantly between the two groups, even after adjustment for potential confounders (nationality and parity). Table 2 illustrates the details of maternal and neonatal outcomes in the two groups.

**Table 2:** Maternal and neonatal outcomes according to the two screening strategies

	Group A	Group B	OR (95%CI)	*p*-value	aOR (95% CI)^a^
Gestational Diabetes Mellitus	*n* = 3496	*n* = 2555			
	119 (3.4%)	416 (16.28%)	5.51 (4.46–6.81)	< 0.001	5.53 (4.48–6.84)
Primary Cesarean section	*n* = 3135	*n* = 2321			
	386 (12.31%)	320 (13.79%)	1.13 (0.97–1.33)	0.109	1.07 (0.91–1.26)
Gestational hypertensive disorders	*n* = 3496	*n* = 2555			
	101 (2.89%)	73 (2.86%)	0.98 (0 .72–1.34)	0.942	0.93 (0.69–1.27)
Preterm birth	*n* = 3496	*n* = 2555			
	228 (6.52%)	162 (6.43%)	0.97 (0.78–1.19)	0.777	0.97 (0 .79–1.2)
5′ Apgar score < 7	*n* = 3489	*n* = 2547			
	76 (2.18%)	56 (2.2%)	1.009 (0.71–1.43)	0.957	0.98 (0.69–1.40)
Transfer to NICU	*n* = 3404	*n* = 2547			
	277 (8.14%)	208 (8.17%)	1.003 (0.83–1.21)	0.968	0.97 (0.81–1.18)
Neonatal hypoglycaemia	*n* = 392	*n* = 438			
	65 (16.58%)	90 (20.55%)	1.3 (0.91–1.85)	0.144	1.37 (0.96–1.97)
Shoulder dystocia	*n* = 3489	n = 2547			
	64 (1.83%)	56 (2.20%)	1.2 (0.83–1.72)	0.317	1.19 (0.83–1.72)
LGA	n = 3496	*n* = 2555			
	433 (12.39%)	276 (10.81%)	0.85 (0.73–1.006)	0.059	0.87 (0.74–1.03)
Macrosomia	*n* = 3496	*n* = 2555			
	319 (9.12%)	198 (7.75%)	0.83 (0 .69–1.006)	0.059	0.85 (0.71–1.03)

^a^adjusted for nationality and parity

## Discussion

To our knowledge, this is the first study in Europe that compares the prevalence of GDM obtained with the IADPSG one-step criteria to the prevalence obtained with the two-step approach as recommended by the American Diabetes Association (ADA) in 2003 (defined as a 50 g CGT followed by a 75 g OGTT), instead of the classical Carpenter an Coustan (CC) criteria (where the GCT is followed by a 100 g OGTT [16]).

In our study we have observed that, once our institution introduced IADPSG criteria, the prevalence of GDM increased dramatically from 3.4 to 16.3%, which translated into an OR of 5.51. The OR did not vary in a meaningful way even after we took into consideration and corrected for the evolution of the characteristics in our population, which showed a significant increase of the proportion of primiparous and foreign patients. The observed GDM prevalence in Group B was similar to the one reported in the original HAPO study cohort [5].

This increase of GDM prevalence when shifting from two-step to IADPSG criteria has been consistently reported across studies, since IADPSG criteria are designed to identify milder cases of GDM. The magnitude of the increase varies in different reports [7–11, 17]. One randomized controlled trial showed a non-statistically significant difference in the prevalence of GDM [18], but it was probably due to the small sample size.

We expected a higher rate of GDM in Group A, similar to those reported by other authors who investigated GDM prevalence in the Belgian French-speaking Community, which varied from 5.2% [19] to 8% [11]. However, in both publications, the two-step GDM screening was done with classical Carpenter and Coustan criteria, which indicates that screening with a 75 g diagnostic OGTT might have been even more restrictive in the diagnosis of GDM than with a 100 g OGTT.

In our methodology, we chose to calculate GDM prevalence only on the analysis of the screening tests that we could retrieve, and not on the flagging of a patient as GDM in the patient’s file. The rationale for this decision was that we feared underreporting of GDM, since the flagging was possible but not compulsory. Surprisingly, we observed that in group A flagged GDM patients were 5.53% (data not shown). This phenomenon has been also described by Kong et al. [10] where they assumed that women screened with Carpenter and Coustan criteria with borderline results might have been flagged as GDM.

The advantage of introducing the IADPSG criteria is that we expect a reduction, in the screened population, of obstetric adverse outcomes such as GHD, macrosomia, LGA newborns, and potentially a lower rate of CS and shoulder dystocia.

A secondary analysis of the HAPO study [20] concerning untreated participants in North America demonstrated that the extra women who were classified as GDM by IADPSG criteria, had worse neonatal outcomes than those who were screened negative (and better outcomes than those who were screened positive by CC criteria). This was observed for the outcomes related to adiposity of the newborn (evaluated by macrosomia and LGA), but also for GHD.

This secondary analysis confirmed the argument underlying the HAPO study, namely that GDM-linked obstetric complications correlate in a continuous fashion with levels of glycaemia [5] - but it didn’t say if screening and treating milder cases of GDM improve overall outcomes.

Although theoretically treating milder cases of GDM should lead to better obstetric outcomes [20–23], there is yet no conclusive evidence that this improvement exists when applying the criteria in real-life clinical settings.

In our cohort, even if we diagnosed almost five times more cases of GDM using the IADPSG criteria, and therefore the impact on outcomes should be relevant, the prevalence of obstetric complications most frequently linked to GDM appeared to remain stable. There was however a decrease in the proportion of macrosomia and LGA babies, which fell short of statistical significance.

Similar results were seen in other retrospective studies [7, 8, 10], in particular the one by Ortio et al., where GDM prevalence went from 8 to 23% [11].

In contrast, a significant decrease of poor pregnancy outcomes (among which gestational hypertension and LGA) has been observed after diabetological care of the extra women classified as GDM by IADPSG criteria [9] . It is interesting to notice that in the article by Duran et al. [9], the glycemic target towards which GDM patients were educated was a fasting glucose level < 90 mg/dl and a 1 h post-meal glucose < 120 mg/dl, which is lower than the recommended thresholds in our population.

It is important to note that, to this day, an adequately powered prospective randomized trial comparing maternal and neonatal outcomes in pregnancies screened with the CC and the IADPSG criteria, has not been carried out.

Moreover, the increase in prevalence that occurs after the implementation of the IADPSG screening strategy is bound to put a strain in the pathway of care of GDM positive patients, and may lead to a potential overmedicalization of such pregnancies. This is why the cost effectiveness of the IADPSG screening strategy is still object of debate [24].

In our view, even if applying the IADPSG criteria should decrease obstetric complications linked to GDM, the number needed to screen in order to detect a positive result in maternal and neonatal outcomes may be high.

The decrease in obstetric complications, though, depends on many variables other than the screening strategy alone. These variables include screening and treatment acceptability by patients [25], glycemic targets, adherence to treatment, and the independent influence of maternal BMI [6]. The strength of obstetrics and endocrinological care coordination might also have to be explored carefully.

This study has several limitations: there was a significant proportion of patients who delivered at Erasme hospital for whom we could not retrieve GDM screening results in our files (32% in group A and 24% in group B), and this could have had an impact on our estimate of GDM prevalence in the population delivering in our hospital. Nonetheless we observed that the majority of individuals for whom we have no results are those who were followed up in private practices and came to Erasme only for delivery. In this case we could say that the reported GDM prevalence is that of patients that are routinely followed up at Erasme.

Another limitation is the retrospective nature of our study, which means that there are many variables that we could not control for. In particular, we could not control for how the adherence to treatment has changed in the two time periods. This potential bias would have been interesting to factor in since the increase of GDM prevalence following the switch in screening strategy has surely put a burden on the pathway of care for GDM positive patients. In addition to that, our data on the history of GDM for multiparous patients and for family history of T2DM were not reliable and were, therefore, not reported. We cannot exclude the role of these potential confounding factors in the evolution of the prevalence of GDM in our population.

We conclude that in our population, with the current care for GDM positive patients, screening and treating milder cases of GDM does not seem to translate into improved obstetric outcomes. We do observe, though, a tendency to a smaller proportion of LGA babies and macrosomia in the group screened with IADPSG criteria.

Further research will have to be carried out, in particular an adequately powered prospective experimental study is needed to establish if applying the IADPSG criteria decreases the frequency of adverse obstetric outcomes.

## Conclusions

In our population the introduction of IADPSG screening criteria has increased the prevalence of GDM without having a statistically significant impact on pregnancy outcomes. We observe a non-statistically significant decrease in LGA babies and macrosomia.

## Data Availability

The datasets used and analysed during the current study are available from the corresponding author on reasonable request.

## Abbreviations

ADA: American Diabetes Association
AUDIPOG: Association des Utilisateurs de Dossier Informatisés en Pédiatrie, Obstetrique et
BMI: Body Mass Index
CC: Carpenter and Coustan
CS: Cesarean Section
FPG: Fasting Plasma Glucose
GCT: Glucose Challenge Test
GDM: Gestational Diabetes Mellitus
GHD: Gestational Hypertensive Disorders Gynécologie
HAPO: Hyperglycemia and Adverse Pregnancy Outcome
IADPSG: International Association of Diabetes in Pregnancy Study Group
LGA: Large for Gestational Age
NICU: Neonatal Intensive Care Unit
OGTT: Oral Glucose Tolerance Test
OR: Odds Ratio
pCS: primary Cesarean Section
T2DM: Type 2 Diabetes Mellitus
ULB: Université Libre de Bruxelles
WG: Weeks Gestation
